# Assessing the adequacy of self-reported alcohol abuse measurement across time and ethnicity: cross-cultural equivalence across Hispanics and Caucasians in 1992, non-equivalence in 2001–2002

**DOI:** 10.1186/1471-2458-9-60

**Published:** 2009-02-19

**Authors:** Adam C Carle

**Affiliations:** 1 Department of Psychology, University of North Florida, 1 UNF Drive, Jacksonville, FL 32224, USA

## Abstract

**Background:**

Do estimates of alcohol abuse reflect true levels across United States Hispanics and non-Hispanic Caucasians, or does culturally-based, systematic measurement error (i.e., measurement bias) affect estimates? Likewise, given that recent estimates suggest alcohol abuse has increased among US Hispanics, the field should also ask, "Does cross-ethnic change in alcohol abuse across time reflect true change or does measurement bias influence change estimates?"

**Methods:**

To address these questions, I used confirmatory factor analyses for ordered-categorical measures to probe for measurement bias on two large, standardized, nationally representative, US surveys of alcohol abuse conducted in 1992 and 2001–2002. In 2001–2002, analyses investigated whether 10 items operationalizing DSM-IV alcohol abuse provided equivalent measurement across Hispanic (*n *= 4,893) and non-Hispanic Caucasians (*n *= 16,480). In 1992, analyses examined whether a reduced 6 item item-set provided equivalent measurement among 834 Hispanic and 14,8335 non-Hispanic Caucasians.

**Results:**

In 1992, findings demonstrated statistically significant measurement bias for two items. However, sensitivity analyses showed that item-level bias did not appreciably bias item-set based alcohol abuse estimates among this cohort. For 2001–2002, results demonstrated statistically significant bias for seven items, suggesting caution regarding the cross-ethnic equivalence of alcohol abuse estimates among the current US Hispanic population. Sensitivity analyses indicated that item-level differences *did *erroneously impact alcohol abuse rates in 2001–2002, underestimating rates among Hispanics relative to Caucasians.

**Conclusion:**

1992's item-level findings suggest that estimates of drinking related social or legal problems may underestimate these specific problems among Hispanics. However, impact analyses indicated no appreciable effect on alcohol abuse estimates resulting from the item-set. Efforts to monitor change in alcohol abuse diagnoses among the Hispanic community can use 1992 estimates as a valid baseline. In 2001–2002, item-level measurement bias on seven items did affect item-set based estimates. Bias underestimated Hispanics' self-reported alcohol abuse levels relative to non-Hispanic Caucasians. Given the cross-ethnic equivalence of 1992 estimates, bias in 2001–2002 speciously minimizes current increases in drinking behavior evidenced among Hispanics. Findings call for increased public health efforts among the Hispanic community and underscore the necessity for cultural sensitivity when generalizing measures developed in the majority to minorities.

## 1. Background

Psychological science and its allied disciplines too often stand culturally blind, rarely questioning if concepts and measurements valid in the majority culture demonstrate similar validity among minority communities. Despite the fact that alcohol dependence often leads to greater impairment than alcohol abuse and that some recent arguments call for a single diagnostic category encompassing both,[1] alcohol abuse remains a separate diagnostic category in DSM-IV and embodies a substantial public health problem. For example, recent estimates suggest a DSM-IV alcohol abuse prevalence rate of approximately 4.7% in the US. [2] However, studies note significant differences when comparing the prevalence and comorbidity of alcohol abuse and dependence across Caucasians and cultural minorities. [3,4] Consistent with earlier work,[5,6] these studies show a significantly lower rate of comorbid alcohol disorders among Hispanics as compared to Caucasians. [4] Work also establishes a changing trend of drinking behavior across Caucasians and Hispanics, remaining relatively stable among Caucasians and increasing for Hispanics. [4] These investigations demonstrate etiological and epidemiological differences in alcohol abuse across Caucasians and Hispanics and highlight the need for culturally sensitive public health policy and prevention and intervention efforts, particularly given the presence of health disparities [7-12] and the colossal cost of alcohol abuse to individuals, families, and society. [13,14] Despite this work, research has not adequately explored culture's possible influence on alcohol abuse.

The previous comparisons frequently rest on the *untested *assumption that concepts and measurements reliably and validly estimated among the majority Caucasian culture achieve similar reliability and validity among the Hispanic community. Measurement bias, also labeled differential item functioning (DIF), refers to the possibility that individuals equal in their true levels of alcohol abuse, but from different groups, i.e., Caucasians and Hispanics, do not have identical probabilities of responding to questions concerning their alcohol use. [15] Although studies have established the validity and reliability of standardized alcohol abuse measures and diagnostic criteria in the general population, and provided support for single factor models, [16-26] the role of minority/majority based measurement bias in the instruments used to assess alcohol abuse in the U.S. population goes relatively unexamined.

Modern measurement models, such as confirmatory factor analysis (CFA), offer powerful tools to examine bias. [27,28] Generally, these methods use equations to model item response probabilities and compare the equality of the parameters associated with these models across groups to investigate bias. While investigations of this type have not examined measurement bias and alcohol abuse in recent data, they have examined cultural differences across a number diagnostic measures generally, e.g., dementia, depression, etc. [27] They have shown that bias can attenuate or accentuate group differences, [28-32] lead to inaccurate diagnoses,[28,33-35] and generally decrease reliability and validity. [36-41] Studies have also uncovered bias so profound as to render cross group comparisons virtually impossible. [42-44] Thus, before validly comparing minority and majority groups, we must ask whether the measurements upon which we base comparisons function similarly across groups. [27,45] The field must consider the extent to which observed differences and change reflect true differences or result from a lack of equivalence in the measures used to assess alcohol abuse across populations.

Theoretical and empirical reasons suggest we should suspect measurement bias across Hispanics and Caucasians. [46] Authors have noted differences in the relation between probabilistic thinking and assignment of numbers,[47] differences in acquiescent responses,[48] and differences in language use[49] across Hispanics, Caucasians, and other minorities. A number of authors have also noted that behavioral exemplars describing a psychological construct for the majority may not be appropriate for a minority group, nor do they necessarily include a set of culturally appropriate indicators for minorities. [30,43,44] Hui and Triandis have described sincerity as a cultural value among Hispanics that may lead to measurement bias, positing that the Hispanic culture generally values sincere responses that lead to more ready endorsements of scale end points because the middle of scales often reflect a "don't know", "no opinion", or similar option. Prelow, et al. [44] suggest that for certain behaviors greater levels of a specific problem may be needed before Hispanics willingly acknowledge a problem. McHorney and Fleishman[50] note that survey questions may trigger differential cultural perceptions regarding socially desirable responses and that question wording may impede symptomatology reporting by Hispanics. In sum, we have strong reason to express concern that measurement bias affects the equivalence of measurement across Hispanics and Caucasians generally, and have no reason to exclude alcohol abuse from these suspicions. Indeed, in a recent reexamination of alcohol dependence among a 1992 cohort, Carle [51] found statistically significant measurement bias across Hispanics and non-Hispanic Caucasians. This bolsters concerns about current estimates, particularly as they relate to earlier assessments.

Woefully, a literature review found no published studies examining the validity of alcohol abuse measures across Caucasians and Hispanics in recent or early data. It remains ambiguous whether measurement bias affects epidemiological estimates and research across these groups. This leaves unclear whether recent documentation suggesting differential prevalence and comorbidity of alcohol abuse and discrepant drinking pattern changes reflect true self-reported statuses, measurement bias, or both. As a result, in the current study, I had several goals. I used modern measurement models to examine whether measurement bias exists across Hispanic and non-Hispanic Caucasians on two standardized measures of alcohol abuse in a large, nationally representative survey of alcohol use in the United States conducted in 1992 and in 2001–2002, and, if so, to what extent does it impact estimates of alcohol abuse across non-Hispanic Caucasians and Hispanics at these points in time. I used these results to assess whether descriptions noting recent changes in alcohol abuse ought to receive modification. Should we *increase *or *decrease *current estimates as a function of biased measurement?

## 2. Methods

### 2.1 Participants

#### 2.2.1 1992

Participants (*n *= 14,835; 14,001 non-Hispanic Caucasians and 834 Hispanics) were a subset of the larger 1992 National Longitudinal Alcohol Epidemiologic Study (NLAES),[52] designed and sponsored by the National Institute for Alcohol Abuse and Alcoholism (NIAAA), and fielded by the U.S. Census Bureau. The original sample consisted of 42,862 U.S. adults aged 18 years and older, selected at random from a sample representative of U.S. households nationwide. The complex, multistage design oversampled both the African American population and young adults between the ages of 18 and 29, and had household and sample person response rates of 92% and 97% respectively. Sample weights adjust the data to make it representative of the civilian non-institutionalized population of the US [52] non-Hispanic Caucasians and Hispanics with complete data were included in the current study. In the original design, 14,835 individuals who reported consumption of alcohol in the past 12 months were asked the questions studied here.

#### 2.2.2 2001–2002

Participants (*n *= 21,373; 16,480 non-Hispanic Caucasians and 4,893 Hispanics) were a subset of the larger, publicly available 2001–2002 National Epidemiologic Survey on Alcohol and Related Conditions (NESARC[53]) data designed and sponsored by the National Institute for Alcohol Abuse and Alcoholism (NIAAA) and fielded by the US Census Bureau. The original sample consisted of 43,093 US adults aged 18 years and older representing the non-institutionalized adult US population. The complex, multistage design incorporated the Census 2000/2001 Supplementary Survey (C2SS) and Census 2000 Group Quarters Inventory sampling frame and oversampled African American, Hispanics, and young adults (18 – 24). Sample weights, described in detail elsewhere[53], adjust the data to make it representative of the civilian non-institutionalized population of the US. The NESARC had household and sample person response rates of 89% and 93% respectively[53]. The current study included participants with complete data who reported consumption of alcohol in the past 12 months.

### 2.2 Procedures

For both surveys, experienced Census Bureau interviewers completed direct face-to-face interviews in respondents' homes and recorded information concerning: alcohol consumption and problems, drug use and problems, periods of low mood, utilization of alcohol and drug treatment, alcohol-related physical morbidity, family history of alcoholism, and sociodemographic background characteristics.

### 2.3 Measures

#### 2.3.1 Alcohol Abuse

The DSM-IV [54] identifies alcohol abuse as a maladaptive pattern of alcohol use that occurs in the absence of alcohol dependence and leads to significant impairment or distress, and that demonstrates at least one of the following four criteria: 1) continued use despite a social or interpersonal problem caused or exacerbated by the effects of drinking; 2) recurrent drinking in situations in which alcohol use is physically hazardous; 3) recurrent drinking resulting in a failure to fulfill major role obligations; or 4) recurrent alcohol related legal problems.

#### 2.3.1.1 1992

The Alcohol Use Disorder and Associated Disabilities Interview Schedule (AUDADIS[55]) used in the NLAES uses a total of 6 dichotomous items to operationalize alcohol abuse criteria. The AUDADIS provides a fully structured diagnostic interview schedule that includes modules to measure alcohol and drug use, major mood disorder, substance-related medical conditions, and family history of alcohol and drug use disorders. It generates diagnoses consistent with the several diagnostic classification systems including the Fourth Edition of the DSM (DSM-IV). I used all 6 items operationalizing alcohol abuse criteria. Reliabilities established through independent test-retest meet acceptable standards. [56] Additional studies have also established several types of validity, e.g., construct, criterion, etc. [17,18,22,23,25,26,56-59]

#### 2.3.1.1: 2001–2002

The Alcohol Use Disorder and Associated Disabilities Interview Schedule DSM-IV Version (AUDADIS-IV[19]) used in the NESARC uses a total of 10 dichotomous items to operationalize alcohol abuse criteria. The AUDADIS-IV is an updated version of the AUDADIS used in the NLAES. Like its predecessor, it generates diagnoses consistent with DSM-IV and has demonstrated acceptable psychometric standards. [17,18,22,23,25,26,56-59] I used all 10 items operationalizing alcohol abuse criteria.

#### 2.3.2 Ethnicity

Both the NLAES (1992) and NESARC (2001–2002) coded race using five options: American Indian and Alaska Native; Asian; Black or African American; Native Hawaiian and Other Pacific Islander; and White. A single item allowed Hispanic self-identification. The current study considered individuals non-Hispanic Caucasians if they identified themselves as both White and non-Hispanic and regarded anyone who self-identified as Hispanic a Hispanic.

### 2.4 Analytic Strategy

The current study used confirmatory factor analyses for ordered-categorical measures (CFA-OCM) to probe for bias. CFA-OCM appropriately models the categorical nature of the items[60] and falls within a larger family of latent variable measurement modeling approaches that includes: CFA for continuous measures, multiple indicator multiple cause (MIMIC) models, and item response theory models. Given its strengths relative to the weaknesses of other approaches, the present study adopted CFA-OCM. For example, MIMIC models: require invariant loadings across the groups, a challenging assumption when working with understudied measures like the one here;[61] require invariant factor variances across the groups; lack formal hierarchical invariance tests; and may miss non-uniform bias. [61,62] Additionally, because a MIMIC model would control for the effects of ethnicity, this approach would not allow analyses to examine whether group differences remain after modeling measurement bias, a specific goal of this study. CFA-OCM does not suffer from these issues. With regard to IRT, Takane and de Leeuw[63] demonstrated the functional equivalence of CFA-OCM and 2 parameter IRT models making the choice between them relatively superficial, although estimation procedures can differ in practice. [64] However, IRT modeling procedures include fewer indices relative to CFA,[61,65] and, as a result, CFA-OCM can provide more informed model fit examinations and one can mathematically derive IRT parameter estimates post hoc. For these reasons, the study used CFA-OCM. Unfortunately, few social scientists receive training in these models. As a result, I review them briefly. However, the interested reader should consult Byrne[66], Millsap & Yun-Tien[67], Muthén[68], or Muthén & Christoffersson[60] for detailed reviews.

CFA-OCM indicates a set of equations to describe the relations among a set of ordered-categorical items, suggesting that individuals' item responses are determined by their value on an underlying factor or factors and several measurement parameters. In the CFA-OCM model, loadings, similar to correlations, represent the degree to which an item relates to the factor(s); the greater the value of the factor loading, the greater the relation between the item and the latent variable of interest. The threshold parameters, reflect the ordered-categorical nature of the items. The model assumes that a continuous latent response variate underlies discrete item response categories. If an individual's value on the latent response variate is less than the threshold, they will respond in one category, but, if their value is greater than the threshold, they will respond in at least the next highest category. Intercept parameters give the expected value of an item when the value of the underlying factor(s) is zero, and uniquenesses include sources of variance not attributable to the factor(s), including measurement error. [69]

**Figure 1:**
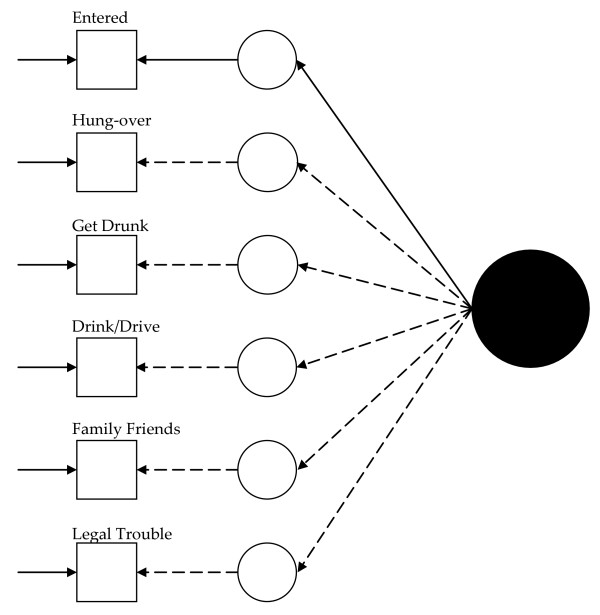
**Path diagram summarizing Model 1, the baseline 1992 alcohol abuse measurement model**. The solid black circle represents the alcohol abuse variable. The small circles represent the continuous latent response variates underlying the dichotomous items (represented by the squares). The arrows from the solid black circle to the smaller circles represent the loadings. The arrows from the small circles to the squares represent the thresholds. The arrows pointing only to the squares represent the uniquenesses. Solid lines represent values constrained to equality across non-Hispanic Caucasians and Hispanics. Dashed lines represent values freely estimated across non-Hispanic Caucasians and Hispanics.

Figure 1 presents a visual representation of this measurement model. The solid black circle represents the latent variable, here alcohol abuse. The small circles represent the continuous latent response variates underlying the dichotomous items (represented by the squares). The arrows from the solid black circle to the smaller circles represent the loadings. The arrows from the small circles to the squares represent the thresholds. Finally, the arrows pointing only to the squares represent the uniquenesses.

In measurement bias studies, researchers examine the equivalence of the measurement parameters across groups. In practice, a series of hierarchically nested models typically test measurement bias. [66,67,69] The method starts with the least restricted measurement model across groups and adds cross-group equivalence constraints in the measurement parameters in a stepwise fashion in later models. Fit indices describe the tenability of the equivalence constraints in a given set of measurement parameters at each step. When these indices suggest untenable constraints, analyses have identified statistically significant measurement bias. Finally, work of this type distinguishes between full and partial measurement invariance. Full measurement invariance implies that an entire set of item parameters achieves equality across the groups, e.g., all of the loadings, thresholds, intercepts, and uniqueness demonstrate equivalence. However, statistically significant measurement bias may result from a limited number of parameters rather than bias across the entire set of item, e.g., a small number of loadings. To investigate this, analysts test a partial measurement invariance hypothesis. This hypothesis constrains some measurement parameters to equality across the groups and allows inequivalence in others. In this way, researchers can fully model cross-cultural differences in measurement bias and examine whether some or all items demonstrate bias.

**Figure 2:**
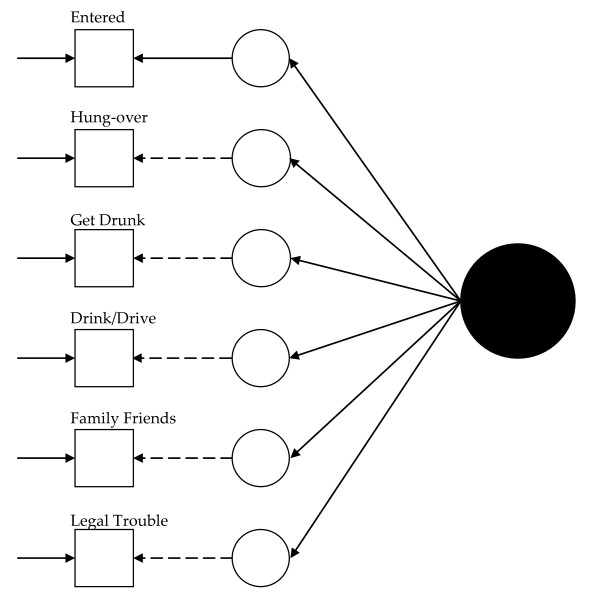
**Path diagram summarizing Model 2, the 1992 alcohol abuse measurement model constraining the loadings to equality across groups**. The solid black circle represents the alcohol abuse variable. The small circles represent the continuous latent response variates underlying the dichotomous items (represented by the squares). The arrows from the solid black circle to the smaller circles represent the loadings. The arrows from the small circles to the squares represent the thresholds. The arrows pointing only to the squares represent the uniquenesses. Solid lines represent values constrained to equality across non-Hispanic Caucasians and Hispanics. Dashed lines represent values freely estimated across non-Hispanic Caucasians and Hispanics.

**Figure 3:**
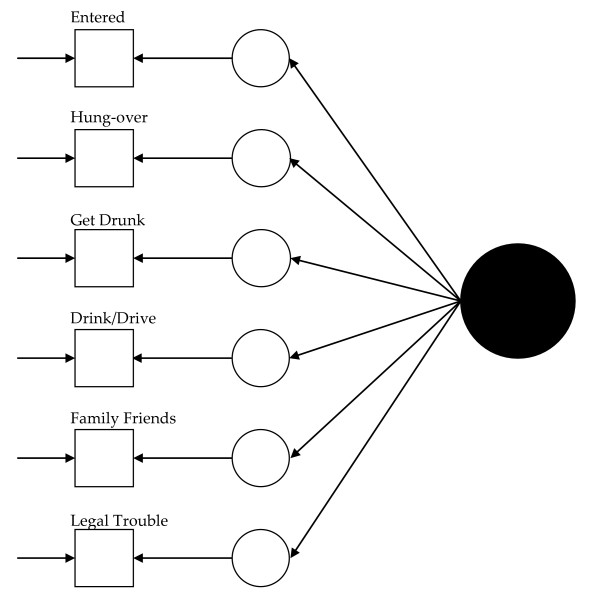
**Path diagram summarizing Model 3a, the 1992 alcohol abuse measurement model constraining the thresholds to equality across groups**. The solid black circle represents the alcohol abuse variable. The small circles represent the continuous latent response variates underlying the dichotomous items (represented by the squares). The arrows from the solid black circle to the smaller circles represent the loadings. The arrows from the small circles to the squares represent the thresholds. The arrows pointing only to the squares represent the uniquenesses. Solid lines represent values constrained to equality across non-Hispanic Caucasians and Hispanics.

**Figure 4:**
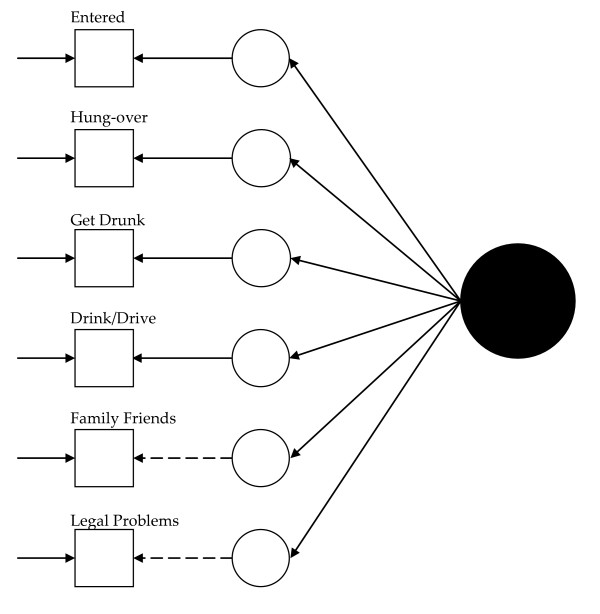
**Path diagram summarizing Model 4, the final 1992 alcohol abuse measurement model**. The small circles represent the continuous latent response variates underlying the dichotomous items (represented by the squares). The arrows from the solid black circle to the smaller circles represent the loadings. The arrows from the small circles to the squares represent the thresholds. The arrows pointing only to the squares represent the uniquenesses. Solid lines represent values constrained to equality across non-Hispanic Caucasians and Hispanics. Dashed lines represent values freely estimated across non-Hispanic Caucasians and Hispanics.

**Figure 5:**
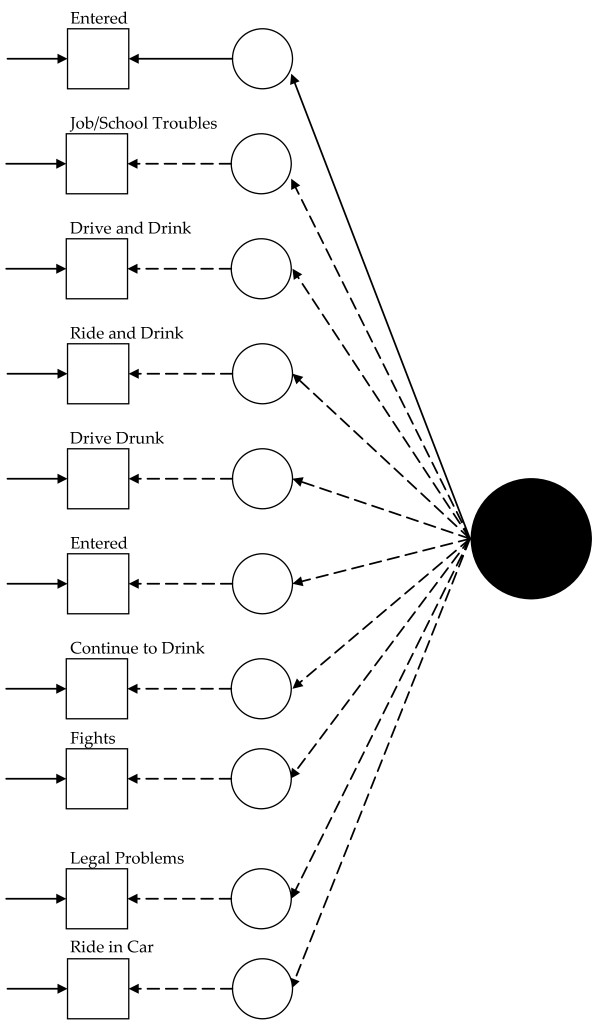
**Path diagram summarizing Model 5, the baseline 2001–2002 alcohol abuse measurement model**. The solid black circle represents the alcohol abuse variable. The small circles represent the continuous latent response variates underlying the dichotomous items (represented by the squares). The arrows from the solid black circle to the smaller circles represent the loadings. The arrows from the small circles to the squares represent the thresholds. The arrows pointing only to the squares represent the uniquenesses. Solid lines represent values constrained to equality across non-Hispanic Caucasians and Hispanics. Dashed lines represent values freely estimated across non-Hispanic Caucasians and Hispanics.

**Figure 6:**
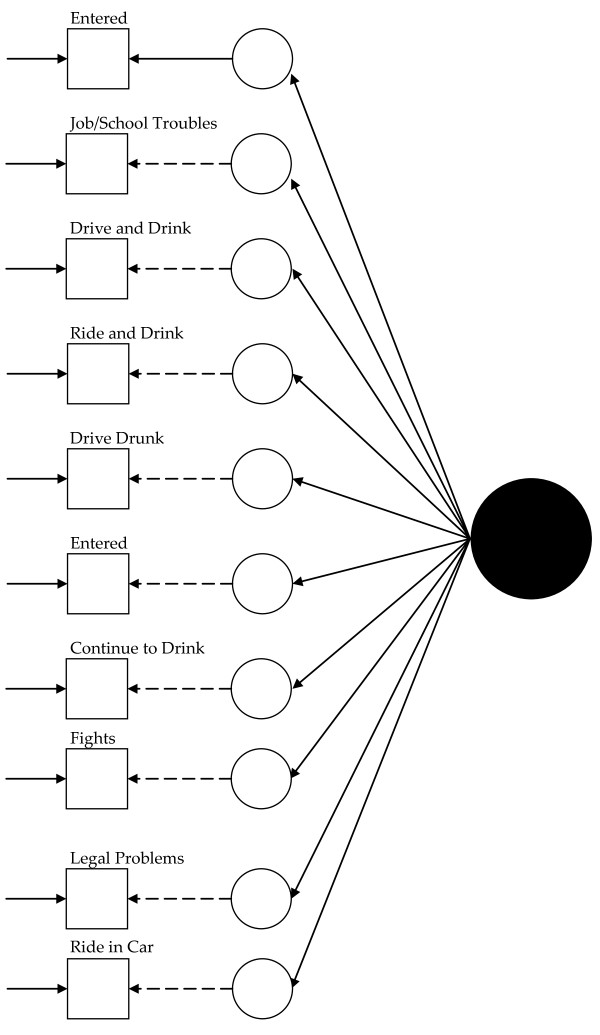
**Path diagram summarizing Model 6a, the 2001–2002 alcohol abuse measurement model constraining the loadings to equality across groups**. The small circles represent the continuous latent response variates underlying the dichotomous items (represented by the squares). The arrows from the solid black circle to the smaller circles represent the loadings. The arrows from the small circles to the squares represent the thresholds. The arrows pointing only to the squares represent the uniquenesses. Solid lines represent values constrained to equality across non-Hispanic Caucasians and Hispanics. Dashed lines represent values freely estimated across non-Hispanic Caucasians and Hispanics.

**Figure 7:**
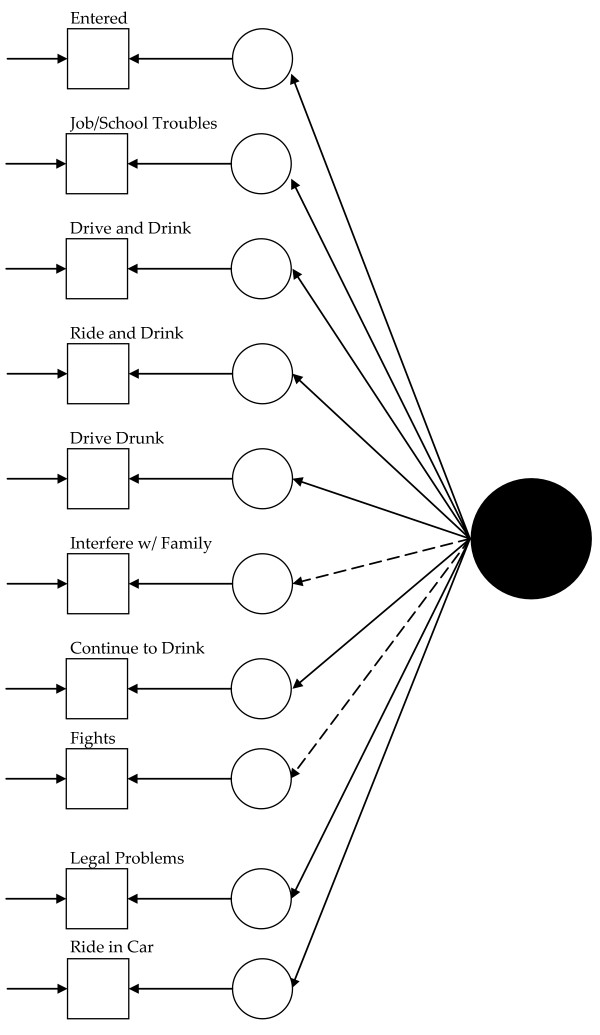
**Path diagram summarizing Model 7a, the 2001–2002 alcohol abuse measurement model constraining the thresholds to equality across groups**. The small circles represent the continuous latent response variates underlying the dichotomous items (represented by the squares). The arrows from the solid black circle to the smaller circles represent the loadings. The arrows from the small circles to the squares represent the thresholds. The arrows pointing only to the squares represent the uniquenesses. Solid lines represent values constrained to equality across non-Hispanic Caucasians and Hispanics. Dashed lines represent values freely estimated across non-Hispanic Caucasians and Hispanics.

**Figure 8:**
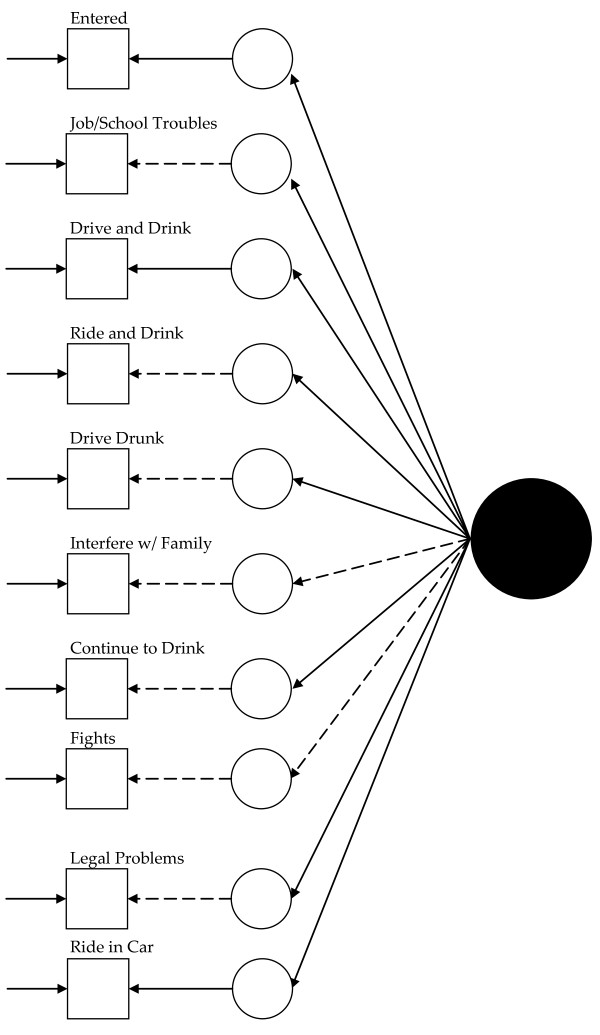
**Path diagram summarizing Model 8, the final 2001–2002 alcohol abuse measurement model**. The small circles represent the continuous latent response variates underlying the dichotomous items (represented by the squares). The arrows from the solid black circle to the smaller circles represent the loadings. The arrows from the small circles to the squares represent the thresholds. The arrows pointing only to the squares represent the uniquenesses. Solid lines represent values constrained to equality across non-Hispanic Caucasians and Hispanics. Dashed lines represent values freely estimated across non-Hispanic Caucasians and Hispanics.

Visually, for the least restricted model in the 1992 data, this would mean fitting a model like that presented in Figure 1 for each group and allowing the measurement parameters to vary across the groups (excepting those constrained to equality for statistical identification, see below). Thus, in Figure 1, dashed lines represent measurement parameters allowed to vary across groups and the solid lines represent measurement parameters constrained to equality across the groups. As Figure 2 shows, the model constraining the loadings across the groups has solid black lines from the latent variable (solid circle) to the latent response variates, indicating that the loadings have been constrained to equality. Figures 3 and 4 continue the visual representation for the 1992 data. Figures 5, 6, 7, and 8 illustrate the models for the 2001–2002 data.

The current investigation adopted this approach and conducted all analyses using M*plus*, a program capable of appropriately handling complex survey data, its theta parameterization and robust weighted least squares (WLSMV) estimator (M*plus *4.2)[64]. Analyses examined measurement invariance following the hierarchical method described above and in detail by Millsap and Yun-Tien[67]. A priori, the studied adopted preferred fit index levels suggested by Hu and Bentler[70,71], Muthén and Muthén[64], Steiger[72], and Cheung and Rensvold:[73] root mean square error of approximation (RMSEA) values less than 0.05; comparative fit index (CFI), Tucker-Lewis Index (TLI), and Gamma Hat values greater than 0.95; and McDonald's noncentrality index (NCI) values greater than 0.90. Fit evaluation focused on the index set. Models included means and covariances at each step and statistical identification conformed to Millsap and Yun-Tien's[67] description. Consistent with arguments for more stringent error control in modeling,[74,75] an α of 0.01 was adopted a priori.

## 3. Results

### 3.1 Measurement Bias

#### 3.1.1 1992

Previous work guided initial model selection. [25,26] Consistent with Muthén and colleague's work suggesting the adequacy of a single factor alcohol abuse model[25,26], analyses tested the fit of a baseline single factor model across the Hispanic and non-Hispanic Caucasian groups. Second, preceding work [76-78] recommended using the "entered into dangerous situation after drinking" item as an item free of bias across these groups. Thus, for statistical identification, the baseline model: fixed the factor mean at zero and variance at one for the non-Hispanic Caucasian group, constrained item intercepts to zero in each group, constrained the loading for the "entered " item to equality across the groups, constrained the threshold for the "entered" item to equality across the groups, and fixed the uniquenesses to a value of one in each group. It included no covariates.

Model 1 (see Figure 1) tested the cross-group fit of a single factor model. Fit indices suggested Model 1 fit well (RMSEA = 0.034, CFI = 0.99, TLI = 0.98, χ^2 ^= 141.33, 15, *n *= 14,001, *p *< 0.01). Model 2 (see Figure 2) retained Model 1's constraints, constrained the loadings to equality, and uncovered no biased loadings (RMSEA = 0.028; CFI = 0.99; TLI = 0.99; χ^2 ^= 118.20, 17, *n *= 14,001, *p *< 0.01; Δχ^2 ^= 7.31, 4, *n *= 14,001, *p = *0.12). Model 3a (see Figure 3) retained Model 2's restraints and constrained the thresholds. A statistically significant Δχ^2 ^demonstrated bias: Δχ^2 ^= 16.78 (5, *n *= 14,001, *p *< 0.01). For "*...causing trouble with family/friends*", and "*...legal problems*", the constraint overestimated Hispanics' thresholds. Model 3b, allowing partial invariance for these two thresholds, fit well (RMSEA = 0.027; CFI = 0.99; TLI = 0.99; χ^2 ^= 129.85, 20, *n *= 14,001, *p *< 0.01; Δχ^2 ^= 7.16, 3, *n *= 14,001, *p *= 0.07). Analyses next compared the fit of a model (4) with free uniquenesses to the equivalent uniquenesses model. Model 4 (see Figure 4) fit well (RMSEA = 0.032; CFI = 0.99; TLI = 0.99; χ^2 ^= 134.43, 16, *n *= 14,001, *p *< 0.01). Constraining the uniquenesses uncovered no biased uniquenesses (RMSEA = 0.027; CFI = 0.99; TLI = 0.99; χ^2 ^= 129.85, 20, *n *= 14,001, *p *< 0.01; Δχ^2 ^= 11.2, 5, *n *= 14,001, *p *= 0.05). Given the final set of fit indices, analyses rejected the fully invariant measurement model and specified a 1992 model with partial invariance in the loadings and thresholds in its place. Table 1 summarizes the final estimates across Hispanics and non-Hispanic Caucasians in 1992 and Figure 4 presents the model visually.

**Table 1:** Partially Invariant Alcohol Abuse Measurement Model Across Hispanics and Non-Hispanic

	Loadings		Thresholds		Proportion Endorsing Symptom	
Abbreviated Item						
Labels	Caucasians	Hispanics	Caucasians	Hispanics	Caucasians	Hispanics

Hung-over During Important Activities	2.00	2.00	-3.96	-3.96	0.038	0.043

Get Drunk During Important Activities	1.67	1.67	-3.62	-3.62	0.031	0.034

Drink And Drive	1.30	1.30	-1.94	-1.94	0.119	0.112

Enter Hurtful/Harmful Situations While Drinking	1.23	1.23	-2.69	-2.69	0.046	0.034

Continue To Drink Causing Trouble with Family and Friends	1.27	1.27	-3.13	-2.79	0.026	0.041

Legal Problems Because of Drinking	0.97	0.97	-3.05	-2.80	0.014	0.022

Caucasians in 1992 (Uniquenesses Constrained to 1 for both groups)

To examine the possibility that the choice of the "entered" item as an anchor might influence the final model's results and interpretation, I iterated a set of analyses using the "riding in car" item that exhibited no bias in these analyses as an anchor. These analyses arrived at the exact same partial measurement invariance model as those using the "entered" item and minimize concerns that analyses using a different item anchor diverge from these.

#### 3.1.2 2001–2002

As above, previous work guided initial model selection,[25,26] and analyses tested the fit of a baseline single factor model across the Hispanic and non-Hispanic Caucasian groups. For statistical identification, the baseline model: fixed the factor mean at zero and variance at one for the non-Hispanic Caucasian group, constrained item intercepts to zero in each group, constrained the loading for the "entered " item to equality across the groups, constrained the threshold for the "entered" item to equality across the groups, and fixed the uniquenesses to a value of one in each group. It included no covariates. The set of fit indices suggested this model (Model 5) fit the data well (RMSEA = 0.039, CFI = 0.98, TLI = 0.98, McDonald's NCI = 0.98, Gamma Hat = 0.996, and χ^2 ^= 733.91, 43, *n *= 21,373, *p *< 0.01). Given the well fitting model, analyses moved to metric invariance, i.e., equivalence in the loadings. This model (6a) retained restraints in the previous model, constrained the loadings to equality across the groups, and allowed variation in the remaining parameters. The Δχ^2 ^test suggested the presence of statistically significant measurement bias: Δχ^2 ^= 37.88 (8, *n *= 21,373, *p *< 0.01), and the hypothesis of metric invariance was rejected. Modification indices (MIs) and expected parameter change indices (EPCs) suggested that constraining the loadings for the "*drinking interfered with taking care of home or family*" and "*get into physical fight while or after drinking*" items predominantly accounted for the increased misfit. These constraints underestimated the extent to which the items related to alcohol abuse for Hispanics. A partially invariant model (6b) relaxing the equivalence constraint for the items' loadings fit the data well: RMSEA = 0.035, CFI = 0.98, TLI = 0.98. McDonald's NCI = 0.99, Gamma Hat = 0.996, χ^2 ^= 620.69 (44, *n *= 21,373, *p *< 0.01), and Δχ^2 ^= 10.62 (6, *n *= 21,373, *p = *0.10). The hypothesis of partial measurement invariance was not rejected, and analyses moved to examining invariance in the thresholds.

This model (7a) retained the partially invariant restraints in the previous model, constrained the thresholds to equality across the groups, and allowed variation in the remaining parameters. Again, the Δχ^2 ^demonstrated statistically significant measurement bias: Δχ^2 ^= 57.91 (7, *n *= 21,373, *p *< 0.01). The MIs and EPCs suggested that the increased misfit principally resulted from seven items. For the "*drinking interfered with taking care of home or family*", "*more than once drive vehicle after drinking*", and "*get into physical fight while or after drinking*" items, the equality constraint underestimated Hispanics' thresholds. For the "*job or school troubles because of drinking*", "*more than once ride in vehicle while drinking*", "*continue to drink despite causing trouble with family or friends*", and "*get arrested or have legal problems because of drinking*" items, the equality constraint overestimated Hispanics' thresholds. A model allowing partial measurement invariance (7b) for these seven thresholds fit the data well: RMSEA = 0.035, CFI = 0.98, TLI = 0.98, McDonald's NCI = 0.99, Gamma Hat = 0.996, χ^2 ^= 622.93 (45, *n *= 21,373, *p *< 0.01), and Δχ^2 ^= 4.87 (2, *n *= 21,373, *p *= 0.09). The hypothesis of partial measurement invariance in the loadings and thresholds was not rejected and analyses moved to the uniquenesses. Analyses next compared the fit of a model (8) with free uniquenesses to the equivalent uniquenesses model. Model 8 fit well (RMSEA = 0.038, CFI = 0.98, TLI = 0.98, McDonald's NCI = 0.98, Gamma Hat = 0.996; χ^2 ^= 740.34, 44, *n *= 21,373, *p *< 0.01). Constraining the uniquenesses uncovered no biased uniquenesses (RMSEA = 0.035, CFI = 0.98, TLI = 0.98, McDonald's NCI = 0.99, Gamma Hat = 0.996; χ^2 ^= 622.93, 45, *n *= 21,373, *p *< 0.001; Δχ^2 ^= 9.738, 6, *n *= 21,373, *p *= 0.14). Given the final set of fit indices, analyses rejected the fully invariant measurement model and specified a 2001–2002 model with partial invariance in the loadings and thresholds in its place. Table 2 summarizes the final estimates across Hispanics and non-Hispanic Caucasians in 2001–2002.

**Table 2:** Partially Invariant Alcohol Abuse Measurement Model across Hispanics and Non-Hispanic Caucasians in 2001–2002 (Uniquenesses Constrained to 1 for both groups)

	Loadings		Thresholds		Proportion Endorsing Symptom	
Abbreviated Item Labels	Caucasians	Hispanics	Caucasians	Hispanics	Caucasians	Hispanics

Drinking Interfered with Taking Care of Home or Family	1.27	2.31	-3.97	-5.78	0.007	0.008

Job or School Troubles Because of Drinking	1.43	1.43	-4.58	-4.25	0.004	0.006

More Than Once Drive Vehicle While Drinking	1.91	1.91	-3.18	-3.18	0.070	0.056

More Than Once Ride In Vehicle While Driver was Drinking	2.29	2.29	-3.25	-2.99	0.097	0.099

More Than Once Drive Vehicle After Too Much to Drink	1.60	1.60	-3.15	-3.25	0.048	0.036

Entered Into Dangerous Situation After Drinking	1.32	1.32	-3.17	-3.17	0.028	0.021

Continue to Drink Despite Causing Trouble With Family or Friends	1.47	1.47	-4.00	-3.58	0.012	0.017

Get Into Physical Fight While or After Drinking	1.02	1.54	-3.20	-3.66	0.013	0.019

Get Arrested or Have Legal Problems Because of Drinking	1.01	1.01	-3.41	-3.13	0.008	0.011

Ride in Car as Passenger While Drinking	1.96	1.96	-2.69	-2.69	0.110	0.101

As with the 1992, I reiterated a set of analyses using the "riding in car" item as an anchor. These analyses arrived at the exact same partial measurement invariance model as those using the "entered" item and minimize concerns that analyses using a different item anchor diverge from these.

### 3.2 Impact

Because statistically significant item level criteria do not always translate into meaningful or practical differences on scale scores, a sensitivity analysis examined the extent to which the statistically significant bias impacted estimates. No gold standard exists for evaluating DIF's impact, especially with ordered-categorical models. [79] In light of this, a number of authors argue for and have shown the utility of conducting analyses that compare the direction and size of mean differences resulting from a fully invariant model ignoring observed measurement bias to those resulting from the model incorporating measurement bias to evaluate impact. [80-82] Changes in mean differences reflect impactful bias. Analyses adopted this approach.

#### 3.2.1 1992

In the invariant model, non-Hispanic Caucasians' mean equaled zero (a function of statistical identification) and Hispanics' mean equaled -0.09 (*z *= -0.82, *p *= 0.41). Under the partially invariant model, this pattern persisted (*M*_*Caucasians *_= 0.00, *M*_*Hispanics *_= -0.05, *z *= -0.44, *p *= 0.66), suggesting little impact. [62,83] Failing to incorporate measurement bias did not affect mean estimates and cross-group comparisons.

#### 3.2.2 2001–2002

In the fully invariant model, non-Hispanic Caucasians had a group mean of zero (a function of statistical identification) and Hispanics had a group mean of 0.49 significantly greater than zero (*M*_*Caucasians *_= 0.00, *SD*_*Caucasians *_= 1, *M*_*Hispanics *_= 0.49, *SD*_*Hispanics *_= 1.26, *z *= 3.39, *p *< 0.01). For these items, higher values reflect less use (1 = "Yes", 2 = "No"). Under the partially invariant model, non-Hispanic Caucasians' and Hispanics' means did not differ significantly (*M*_*Caucasians *_= 0.00, *SD*_*Caucasians *_= 1, *M*_*Hispanics *_= 0.07, *SD*_*Hispanics *_= 0.98, *z *= 1.29, *p *= 0.10). Thus, failing to incorporate statistically significant measurement bias: 1) meaningfully impacts mean estimates and cross-group comparisons, 2) overestimates differences between the groups, and 3) underestimates Hispanics' true use levels. Table 3 completely summarizes these estimates.

**Table 3:** Sensitivity Analyses Presenting Changes in Means across Models Ignoring Measurement Bias (Fully Invariant) and Allowing Measurement Bias (Partially Invariant)

	1992				2001–2002			
	Mean (SD)		*z*	*p*	Mean (SD)		*z*	*p*

	Caucasian	Hispanic			Caucasian	Hispanic		

Fully Invariant	0.00 (1.00)	-0.09 (0.96)	-0.82	0.41	0.00 (1.00)	0.49 (1.26)	3.39	< 0.01

Partially Invariant	0.00 (1.00)	-0.05 (0.95)	-0.44	0.66	0.00 (1.00)	0.07 (0.98)	1.29	0.10

## 4. Discussion

How well does the field measure and estimate change in alcohol abuse among Hispanics in the US? In this study, I sought an answer. First, I examined whether statistically significant, impactful measurement bias presented across Hispanic and non-Hispanic Caucasians on a standardized, six-item measure of DSM-IV alcohol abuse in a nationally representative 1992 US sample. Confirmatory factor analysis for ordered-categorical measures (CFA-OCM) uncovered two biased items. These items addressed drinking related troubles with family and friends and whether individuals experienced drinking related legal problems. Bias resulted in differential reporting tendencies at similar levels of alcohol abuse. Relative to non-Hispanic Caucasians, Hispanics needed to experience fewer "*trouble[s] with family and friends*" and fewer "*legal problems*" to say yes. However, given that partial measurement invariance does not by default lead to biased observed scores,[38,84] nor do statistically significant criteria necessarily translate into meaningful differences[38] I also investigated whether item-level bias affected 1992 item-set alcohol abuse estimates. A sensitivity analysis compared the size and direction of mean differences across a model proceeding as if bias didn't present and a model incorporating measurement bias. This comparison examined whether analyses conducted ignoring measurement bias would diverge from those incorporating bias. These analyses revealed that item-level differences minimally affected item-set alcohol abuse estimates. In other words, do 1992 cross-ethnic alcohol abuse estimates provide valid baseline estimates? Yes.

Second, to better evaluate recent alcohol abuse estimates and differential change in alcohol abuse across time, I examined whether statistically significant measurement bias existed across Hispanic and non-Hispanic Caucasians on a standardized, 10 item measure of DSM-IV alcohol abuse in a recent (2001–2002), large, nationally representative survey of US alcohol use. This addressed whether statistically significant bias impacted the validity of *current *alcohol abuse estimates across Hispanics and non-Hispanic Caucasians. CFA-OCM demonstrated the presence of statistically significant, impactful measurement bias for seven of ten items. These items addressed drinking related legal problems, physical fights, job or school troubles, troubles with family and friends, and drinking related interference with taking care of the home or family, as well as whether individuals drove vehicles after drinking too much and whether they rode in vehicles as passengers while drinking. Differences in responses to these items underestimated rates of alcohol abuse among 2001–2002 Hispanics as compared to non-Hispanic Caucasians. In other words, how valid are current estimates of alcohol abuse across Hispanics and non-Hispanic Caucasians? Not valid enough.

Bias in the loadings showed that two problems better predicted alcohol abuse for Hispanics than they did for non-Hispanic Caucasians; "*drinking related interference with taking care of the home*" and "*physical fights while or after drinking*". Endorsements of these items more closely tied to alcohol abuse for Hispanics than non-Hispanic Caucasians; one would have more faith that Hispanics' item responses reflected alcohol abuse as opposed to some other influence. Bias in the thresholds demonstrated differential reporting tendencies at similar levels of alcohol abuse for seven items. CFA-OCM assumes a continuous latent variate underlies observed yes/no responses and that a threshold determines responses, thus, if an individual's level of the variate is less than the threshold, they answer yes. If not, they answer no. In this study, Hispanics were less likely to endorse several items. Compared to non-Hispanic Caucasians, they needed to experience more "*drinking related interference with taking care of the home or family*", more "*physical fights while or after drinking*", or "*drive [a] vehicle after drinking*" more to say yes. Four items saw a reversed pattern. As a function of alcohol abuse, Hispanics more readily endorsed "*drinking related legal problems*", "*drinking related trouble with family or friends" *and "*drinking related job or school troubles*". Also, Hispanics needed to "*ride in a vehicle as passengers while drinking*" less frequently before upholding the item.

Taken together, these findings present strong evidence that Hispanics in the US currently respond to several items operationalizing alcohol abuse criteria differently than non-Hispanic Caucasians and they call into doubt the cross-cultural equivalence of alcohol abuse measurement across these groups, especially given that the 1992 analyses with a different cohort and reduced item set also identified problems with the "*legal problems" *and "*trouble with family and friends*" items. Moreover, unlike 1992, acknowledging and incorporating measurement bias in the 2001–2002 model lead to *increased *mean reporting levels. Observed scores incorrectly estimate alcohol abuse and fail to provide cross-culturally valid measurement in 2001–2002. Relative to non-Hispanic Caucasians, these findings suggest greater levels of alcohol abuse among Hispanics than previously reported. Given that 1992 estimates do provide a valid baseline, not only is alcohol abuse increasing alarmingly among Hispanics,[3,4] it increases at a greater rate than suspected.

This investigation provides strong evidence that measurement bias presents across Hispanics and non-Hispanic Caucasians when measuring alcohol abuse. A number of mechanisms may *simultaneously *result in this bias, particularly given bias' non-uniform distribution (e.g., some items were more difficult to endorse, others were easier). For example, research notes cultural differences in social desirability and the extent to which Hispanics see psychiatric symptoms as undesirable. [50] Hui and Triandis[85] note that cultural differences in sincerity may influence Hispanic responses. Language skills and socioeconomic variability may also differentially affect responses across these groups. [50] Additionally, the findings may not represent error, but rather accurately reflect fundamental differences in alcohol abuse patterns across non-Hispanic Caucasians and Hispanics. Each of these influences may lead to measurement bias. Future research should seek to elucidate what leads to these differences.

Regardless of bias' source, the findings have implications for public health and clinical practice. First, the US should increase public health alcohol abuse prevention and intervention efforts among Hispanics. Previous work shows that drinking behaviors have increased among this group[4] and the US has devoted resources specifically to address the unique health concerns of minorities[86]. Nevertheless, this study's results demonstrate that alcohol abuse has increased more than suspected and this community deserves more concerted efforts to stem this disease's increase. Second, item level findings suggest that health care interventions aimed at any of the seven criteria that demonstrated bias in 2001–2002 need to consider that responses about these behaviors among Hispanics likely do not reflect problems as they do among non-Hispanic Caucasians. Given similar item-level differences for the "*trouble[s] with family and friends*" and "*legal problems*" items at 1992 and 2001–2002, health care interventions and clinicians should pay particular attention to these two criteria. Ethnic differences exist in the experience and expression of alcohol abuse for Hispanics as compared to non-Hispanic Caucasians. Third, the findings call into question the cross-cultural equivalence of alcohol abuse and highlight the need for culturally sensitive research and prevention and intervention efforts generally. Psychological science should seek the source of this bias and carefully examine the appropriateness of diagnosing and describing cultural minorities using biased items.

Before concluding, the study's strengths and limits deserve review. First, the study focused on ethnic differences. The study did this because a vast body of work examines cross-ethnic differences in alcohol use without regard to other sociodemographic differences and this study intentionally adopted this approach as well to address the validity of these considerable and similarly oriented studies. Probative analyses in the 2001–2002 data examining whether the exact pattern of measurement differences described above present across ethnicity and sex suggested that a relatively similar pattern of measurement bias results when incorporating sex and culture simultaneously, although some minor sex differences present uniquely within culture and some sex differences exhibit exclusively across cultures. However, sample size restrictions resulted in frequent bivariate empty cells, limiting the reliability and interpretability of these analyses. Thus, I report them cautiously here. Second, consistent with other work,[4] the study treated Hispanics as a homogenous cultural group, despite their heterogeneity in America. [87] Analyses could not explore more specifically defined Hispanic groups given their smaller sample sizes within the included Hispanic group, e.g., South American Hispanics *n *= 28, even using Mplus' robust WLSMV estimator. [64] This inability to estimate models for groups this small may miss measurement heterogeneity among Hispanic Americans. Third, the study used a representative sample; it remains unclear whether results would persist in clinical samples.

Finally, these data represent self-reports and may not reflect actual experiences. Without an external gold standard criterion, it remains unclear the extent to which self-reports differ from actual experiences. Additionally, this leaves open the possibility that these questions provide more accurate measurement for Hispanics and poorer measurement for non-Hispanic Caucasians. In other words, without a gold standard, it is possible estimates over-report non-Hispanic Caucasians' alcohol abuse levels rather than under-reporting Hispanics' alcohol abuse. However, given the development of alcohol abuse among the majority non-Hispanic Caucasian population, it seemed reasonable to use non-Hispanic Caucasians as the reference group, as much of the measurement research does. Nevertheless, readers should interpret the findings here with this caution in mind.

These limits leave some issues unaddressed. First, the results of exploratory analyses investigating ethnicity and sex simultaneously highlight the need for future studies with larger sample sizes that could address sociodemographic variability simultaneous to ethnicity. Likewise, by collecting data from larger number of Hispanic individuals, research could examine the equivalence of alcohol abuse measurement within the Hispanic community. A new, larger sample could address this issue. Because clinical samples can differ from community samples,[88] research should examine whether these findings hold in clinical samples. Finally, future research should collect additional data and use an external criterion and examine the extent to which self-reports correspond to the external criterion across non-Hispanic Caucasians and Hispanics. This would clarify whether these findings reflect under-reporting for Hispanics or over-reporting for non-Hispanic Caucasians.

Despite limits, the study has numerous assets. First, it makes an important and unique contribution. A literature review found no studies examining the cross-ethnic measurement equivalence of alcohol abuse in previous or recent US data. Second, it fills this substantial gap using well designed, large, nationally representative samples, alleviating sampling bias and other methodological concerns. Third, it uses modern measurement modeling techniques that allow a sophisticated, precise, and preferred examination of the bias[27]. Finally, it explicitly calls awareness to social science's oft displayed ignorance of cultural variability in measurement.

## 5. Conclusion

In conclusion, results demonstrated the presence of statistically significant measurement bias across Hispanics and non-Hispanic Caucasians for two of six items assessing DSM-IV alcohol abuse in a representative sample of the US in 1992. These item-level differences did not affect alcohol abuse estimates based on the set, though. However, analyses did reveal impactful measurement bias across Hispanics and non-Hispanic Caucasians in a representative sample of the 2001–2002 US for a set of seven items operationalizing DSM-IV alcohol abuse. Results currently suggest caution when diagnosing and estimating rates and levels of alcohol abuse across these groups. Moreover, the study notes that current descriptions *underestimate *the rate of alcohol abuse among Hispanics relative to non-Hispanic Caucasians and that alcohol abuse may be increasing at a greater rate than previously suspected. Finally, these results underscore the need for culturally sensitive research, prevention, and intervention efforts and support the need to empirically question the generalization of psychological findings from the majority group to minority groups in current population data. Summarily, how well does the field currently measure and estimate alcohol abuse across non-Hispanic Caucasians and Hispanics? Not well enough.

## Competing interests

The author declares that they have no competing interests.

## Authors' contributions

Using publicly available data, I worked individually, conducted the literature searches and summaries of previous related work, undertook the statistical analyses, wrote the manuscript, conducted all revisions, and read and approved the final manuscript.

## Pre-publication history

The pre-publication history for this paper can be accessed here:


